# Does Surgical Margin Width Remain a Challenge for Triple-Negative Breast Cancer? A Retrospective Analysis

**DOI:** 10.3390/medicina57030203

**Published:** 2021-02-26

**Authors:** Eduard-Alexandru Bonci, Ștefan Țîțu, Alexandru Marius Petrușan, Claudiu Hossu, Vlad Alexandru Gâta, Morvarid Talaeian Ghomi, Paul Milan Kubelac, Teodora Irina Bonci, Andra Piciu, Maria Cosnarovici, Liviu Hîțu, Alexandra Timea Kirsch-Mangu, Diana Cristina Pop, Ioan Cosmin Lisencu, Patriciu Achimaș-Cadariu, Doina Piciu, Hank Schmidt, Bogdan Fetica

**Affiliations:** 1 11th Department of Oncological Surgery and Gynecological Oncology, “Iuliu Hațieganu” University of Medicine and Pharmacy, 400012 Cluj-Napoca, Romania; bonci.eduard@umfcluj.ro (E.-A.B.); stefan.titu@ymail.com (Ș.Ț.); gata.vlad@umfcluj.ro (V.A.G.); talaeian.morvarid@gmail.com (M.T.G.); teadaria@yahoo.com (T.I.B.); piciuandra@gmail.com (A.P.); cosnarovici_maria@yahoo.com (M.C.); liviu.hitu@umfcluj.ro (L.H.); timeakirsch@gmail.com (A.T.K.-M.); pop.diana@iocn.ro (D.C.P.); pachimas@umfcluj.ro (P.A.-C.); doina.piciu@gmail.com (D.P.); feticab@yahoo.com (B.F.); 2 Department of Surgical Oncology, “Prof. Dr. Ion Chiricuță” Institute of Oncology, 400015 Cluj-Napoca, Romania; apetrusan@gmail.com (A.M.P.); claudiuhossu10@yahoo.com (C.H.); 3 Department of Medical Oncology, “Prof. Dr. Ion Chiricuță” Institute of Oncology, 400015 Cluj-Napoca, Romania; 4 Department of Radiotherapy, “Prof. Dr. Ion Chiricuță” Institute of Oncology, 400015 Cluj-Napoca, Romania; 5 Department of Nuclear Medicine, “Prof. Dr. Ion Chiricuță” Institute of Oncology, 400015 Cluj-Napoca, Romania; 6 Division of Breast Surgery, Tisch Cancer Institute, Mount Sinai Health System, New York, NY 10029, USA; hank.schmidt@mountsinai.org; 7 Icahn School of Medicine at Mount Sinai, New York, NY 10029, USA; 8 Department of Anatomical Pathology, “Prof. Dr. Ion Chiricuță” Institute of Oncology, 400015 Cluj-Napoca, Romania

**Keywords:** triple-negative breast cancer, breast-conserving surgery, local recurrence, surgical margin width, no ink on tumor

## Abstract

*Background and Objectives*: Local and distant relapse (LR, DR) in breast cancer vary according to its molecular subtypes, with triple-negative breast cancer (TNBC) being the most aggressive. The surgical resection margin width (SRMW) for breast-conserving surgery (BCS) has been intensely debated, especially for the aforementioned subtype. The aim of this study was to examine the impact of SRMW on LR following BCS in TNBC patients. *Materials and Methods*: We conducted a retrospective study including all patients with TNBC for whom BCS was performed between 2005 and 2014. *Results*: Final analysis included a total of 92 patients, with a median tumor size of 2.5 cm (range 0–5 cm) and no distant metastasis at the time of diagnosis. A total of 87 patients had received neoadjuvant and/or adjuvant chemotherapy, and all patients had received adjuvant whole-breast radiotherapy. After a median follow-up of 110.7 months (95% CI, 95.23–126.166), there were 5 local recurrences and 8 regional/distant recurrences with an overall LR rate of 5.4%. The risk of LR and DR was similar between groups of patients with several SRMW cut-off values. *Conclusions*: Our study supports a safe “no ink on tumor” approach for TNBC patients treated with BCS.

## 1. Introduction

Globally, breast cancer is the second leading type of diagnosed malignancy, accounting for over two million cases per year [1]. Of these, approximately 10–20% belong to the triple-negative breastcancer (TNBC) subtype [2,3,4,5]. TNBC is characterized by the lack of expression of the estrogen receptor (ER), progesterone receptor (PR), and human epidermal growth factor 2 (HER 2) [6]. The main treatment armamentarium for nonmetastatic TNBC includes surgery, systemic therapy, and radiotherapy.

The last few decades of medical research demonstrated that breast-conserving surgery (BCS) with radiotherapy attains equivalent local control and survival as radical mastectomy does [7,8,9,10,11]. The aim of BCS is to decrease the amount of unnecessary and healthy resected breast tissue, and to increase the quality of life in breastcancer patients. Numerous groups showed that the satisfaction and psychosocial well-being of breastcancer patients is higher among women treated with BCS. Furthermore, postoperative complications are fewer in these patients [12,13,14,15,16]. However, the continuous decrease in size of surgical margin width for BCS has been a source of debate for many years [17,18], with controversy still existing [19,20] in spite of a “no ink on tumor” definition proposed and adopted as the standard surgical margin width (SSO-ASTRO Consensus Guideline on Margins for Breast-Conserving Surgery with Whole-Breast Irradiation in Stage I and II Invasive Breast Cancer) [18].

Major concerns have been raised, especially for TNBC patients due to its higher local recurrence rate (LRR) and distant metastases compared to other molecular subtypes [21,22,23]. Avoiding local recurrence (LR) in breastcancer patients is essential according to a meta-analysis performed by the Early Breast Cancer Trialists’ Collaborative Group. In the aforementioned study, the authors showed that one death could be avoided for every four local recurrences [7]. The “wider is better” belief comes from a series of retrospective studies showing better results for increased surgical margins [24,25,26,27,28]. Nevertheless, the meta-analysis conducted by Houssami et al. [29] on 21 retrospective studies led to the adoption of a “no ink on tumor” consensus for surgical margin width in BCS [18]. Although the authors showed that positive and close margins were significantly associated with a higher LRR, a truly convincing and specific minimal value could not be established to separate a negative margin from a close margin. Furthermore, the meta-analysis did not include subanalyses for each breast molecular subtype. A lack of international consensus for surgical margin width in patients treated with neoadjuvant chemotherapy (NACT) also deepens the aforementioned debate.

To date, only one retrospective study specifically investigated the relationship between surgical margin width and LRR in TNBC patients in the “no ink on tumor” era [30]. Although the study included a large number of patients treated with primary BCS, the authors chose a cut-off value of 2 mm for the surgical margin width and did not provide a detailed analysis for multiple cut-off values.

The main objective of our study was to determine the impact of surgical resection margin width on LR following BCS in patients diagnosed with TNBC in our tertiary-cancer center. Our second objective was to perform subgroup analysis for TNBC patients treated with NACT and BCS. Our data revealed that a “no ink on tumor” consensus [18] seems to be appropriate for TNBC patients treated in our tertiary-cancer center.

## 2. Materials and Methods

### 2.1. Study Population

A retrospective observational study was conducted at ”Prof. Dr. Ion Chiricuță” Institute of Oncology from Cluj-Napoca, Romania (IOCN). This study was approved by the IOCN Ethics Committee (approval number 175 from 22 April 2020) and conducted abiding by the principles stated in the Declaration of Helsinki.

The research included all patients with breast cancer for whom surgery was performed in our institute between 1 January 2005 and 31 December 2014. The patients were identified by searching in the electronic database of histopathological examination results, and were selected if TNBC and BCS were identified.

### 2.2. Definitions (TNBC, Positive Surgical Resection Margin)

TNBC was defined as ductal invasive breast cancer with estrogen receptors (ER) and progesterone receptors (PR) equal to 0%, and HER2 negative (0 or 1+) by immunohistochemistry. If HER2 was 2+, patients with no amplification on fluorescence in situ hybridization (FISH) testing were included in the study.

As our objective was to determine the effect of surgical margin width on LR in the “no ink on tumor” era, the margin was considered positive if “ink on tumor” was present, and patients were excluded from final analysis. Furthermore, to improve the quality of our analysis, several cut-off values for surgical resection margin width were used (2, 3, 4, 5, and 10 mm).

### 2.3. Clinicopathologic and Treatment Variables

The selection of patients was made using the electronic database and medical records from our cancer center. After all patients with TNBC treated with BCS had been identified, we reviewed all medical records with regard to the following aspects: histopathological report of tumor biopsy and surgical specimen, neoadjuvant/adjuvant chemotherapy and radiotherapy records, and follow-up records. After excluding multifocal/multicentric disease, male patients, patients without follow-up data after surgery, patients without whole-breast radiation therapy (WBRT), and those with local recurrence before planned WBRT, we identified 92 patients eligible for final analysis.

Furthermore, we gathered the following data: diagnosis year, menopausal status, age at surgery, clinical stage (cTNM), postoperative stage (pTNM), lymph-node surgical procedure (axillary lymph-node dissection (ALND) or sentinel lymph-node biopsy (SLNB)), number of harvested lymph nodes, number of positive lymph nodes, Nottingham score, lymphovascular invasion (LVI), tumor size on surgical specimen, smallest surgical resection margin, neoadjuvant chemotherapy, time to surgical procedure from NACT, adjuvant chemotherapy (ACT), time from surgical procedure to ACT and/or WBRT, date, status at last follow-up (no disease, alive with recurrence, died of disease, and died of unknown causes), and type of relapse (local, regional, and distant).

All patients underwent lumpectomy and ALND except two for whom lumpectomy and SLNB was performed. Ahead of 2010, the preferred chemotherapy regimen used for TNBC included cyclophosphamide, methotrexate, fluorouracil, and adriamycin. This scheme slowly changed after 2010 to a combination of anthracycline–taxane.

### 2.4. Study Outcomes

After all TNBC cases treated with BCS had been analyzed one by one, the eligible ones were selected with respect to the inclusion and exclusion criteria in order to evaluate the impact of surgical resection margin width on LR, distant recurrence (DR), and overall survival(OS).

Follow-up consultations were studied from the patients’ medical records in order to find the last status of the patient. Follow-up data provided information about local (ipsilateral breast) and regional relapse, and distant metastases.

### 2.5. Statistical Analysis

Statistical software used was IBM SPSS Statistics V22.0 (IBM Corp., Armonk, NY, USA). Pearson χ2 testing was used to assess interactions between variables. Fisher’s exact test was used for expected frequencies <5. An independent-sample t test and Levene’s test for equality of variances were used where appropriate. Survival data were calculated from the date of surgical treatment to the first radiological or clinical progression. Survival curves were estimated using the Kaplan–Meier method and statistically compared using the log-rank test. Median follow-up was quantified using the reversed Kaplan–Meyer method. For multivariate analysis, a Cox model was used. Results were considered significant for two-tailed exact *p* value < 0.05.

## 3. Results

### 3.1. Clinical, Pathological, and Treatment Characteristics

A total of 92 patients with TNBC were included in our study, all of whom had undergone BCS. The median age of the study population was 49.57 years (range 24–74).

Tumor-size range was between 0 and 5 cm, with a median of 2.5 cm. Of the 92 total included breast cancer cases, 85 patients had the smallest resection margin being greater than 2 mm, and 7 patients had the smallest resection margin below or equal to 2 mm; 28.9% had positive nodal status and 27.8% had lymphovascular invasion; 88.7% underwent chemotherapy, of whom 34% had NACT and 64% ACT. All patients received WBRT.

Patient, tumor, and treatment characteristics in relation with surgical margin width status are ilustrated in Table 1. No significant statistical differences were observed in the baseline characteristics between the ≤2 mm and >2 mm resection margin groups.

### 3.2. Local and Distant Recurrence, Kaplan–Meier Recurrence and Survival Outcomes, and Multivariate Analysis

At a median follow-up of 110.7 months (95% CI, 95.23–126.166), we found 5 local recurrences and 8 regional or distant recurrences for the entire study population. LR progresion-free survival (LR-PFS) was 151.56 months (95% CI, 151.56–151.56) for patients with surgical resection margin ≤2 mm, while LR-PFS for patients with surgical resection margin >2 mm was 149.64 months (95% CI, 141.60–157.68; *p* = 0.505). No differences were observed for the risk of distant recurrences (*p* = 0.386) or OS (*p* = 0.583) between the two margin groups.

Multivariate analysis (data not shown) revealed no statistically significant associations between the analyzed factors (age < 50 years, tumor size, lymph-node status, Nottingham score, lymphovascular invasion, and neoadjuvant/adjuvant chemotherapy) and LR, DR, or OS. Moreover, further analyses revealed no differences in terms of LR, DR, and OS for different cut-off values of surgical resection margin width (3, 4, 5, and 10 mm) (Table 2).

### 3.3. NACT Subgroup Analysis

A total of 33 patients with TNBC and NACT were included in subgroup analysis. There were 2 local recurrences and 5 distant recurrences for the entire NACT population. Univariate and multivariate analyses for multiple cut-off values of surgical resection margin width (2, 3, 4, 5, and 10 mm) revealed no differences in terms of LR, DR, and OS. Furthermore, no differences were observed for the risk of LR or DR between the NACT group and the group treated with primary BCS.

## 4. Discussion

Our study included a final number of 92 patients with a median follow-up of 110.7 months (95% CI, 95.23–126.166) and an overall LRR of 5.4%. Although several cut-off values of surgical resection margin were considered (2, 3, 4, 5, and 10 mm), no differences were found in terms of LR and DR between the different groups. Univariate, multivariate, and subgroup analyses peformed for patients with NACT showed a safe “no ink on tumor” approach. Even though other studies also suggested this as a relatively acceptable approach for NACT patients treated with BCS [31,32,33], results should be carefully interpreted due to small-sized cohorts and retrospective designs. Moreover, defining negative margins can be somehow challenging due to possible residual cancers often spread as small foci throughout the initial tumor area [34,35]. While the minimal surgical resection margin width for primary BCS is clearly stated in the SSO-ASTRO Consensus Guideline [18], an adequate margin width definition for patients with NACT is still unknown.

In a meta-analysis that included 14,571 breastcancer patients with a total of 1026 local recurrences, Houssami N. et al. [29] stated that the odds for LR are higher for patients with close versus negative surgical resection margins (*p* < 0.001). However, LR rates did not differ between groups when considering surgical resection margin distance status (1 versus 2 versus 5 mm; *p* > 0.10). Furthermore, the study did not investigate if the surgical margin width of each molecular subtype influences local recurrence. According to many studies, local recurrences and distant recurrences rates seem to be influenced by breast cancer subtype [21,22,23,36]. Nonetheless, Pilewskie M. et al. [30] conducted the first study that specifically investigated the relationship between surgical margin width and LRR in TNBC patients in the “no ink on tumor” era. After analyzing a relatively large cohort of 535 patients, with a median follow-up of 84 months, the study group showed no difference in terms of LR (*p* = 0.11) and DR (*p* = 0.53) for TNBC patients with a surgical resection margin of ≤2 and >2 mm, respectively. The cumulative incidence for the two groups was 4.7% (95% CI 0, 10.0) and 3.7% (95% CI 1.8, 5.5), respectively. However, the study did not provide a detailed analysis for several cut-off values as that of Houssami N. et al. [29] did. Our study confirms a local recurrence rate of around 5% and reiterates the scientific support for “less is more” and better [37]. For the first time, our study analyzes several cut-off values for the smallest surgical resection margin (2, 3, 5, and 10 mm) for TNBC patients with no observed differences in terms of LR and DR between groups (*p* > 0.05).

The meta-analysis performed by Lowery A.J. et al. [21], after including 12,592 breastcancer patients who had undergone BCS (7174) or mastectomy (5418), showed an increased risk of LR for TNBC patients, but no differences in LRR between conservative and radical surgical treatment (OR = 0.83; 95% CI 0.37–1.85)(Table 3). Other studies also suggested that higer LRR for TNBC occur due to its aggressive biology, and are not related to surgical resection margin width [38,39,40]. Therefore, surgical resection margin could play a lesser role for LR, and effective systemic therapy should be the main treatment concern, but the lack of a wide and diverse treatment armamentarium against TNBC makes it hard to tackle when recurrence is diagnosed.

There is no doubt about the benefits of BCS for breast cancer patients, including TNBC patients [12,13,14,15,16,37]. Although almost seven years have passed since the adoption of the SSO-ASTRO Consensus Guideline on Margins for Breast-Conserving Surgery, skepticism still exists among surgeons when it comes to appropriate surgical resection margin width for patients diagnosed with breast cancer, especially those with an aggressive tumor biology. A survey published by DeSnyder S. et al. showed that more than 12% of the 3057 respondents, members of the American Society of Breast Surgeons (ASBrS), would perform re-excision for TNBC with surgical resection margin ≤1 mm [41]. However, current studies reported a change in re-excision rates for patients treated with BCS [42,43,44,45] after the publication of the SSO-ASTRO Consensus Guideline [18]. A cohort of 237 breast cancer patients studied by Bhutiani N. et al. [42] divided patients treated in the pre- and postadoption era of the SSO-ASTRO Consensus Guideline [18]. A decrease of approximately 25% in re-excision rates (*p* < 0.001) and approximately 1000 dollars/patient (*p* < 0.001) was observed in the post-era group. However, several studies showed a major increase in “negative margin” results for BCS (due to the new definition of “no ink on tumor”), but a very small drecrease in re-excision rates, far from the expected results of the SSO-ASTRO Consensus Group [44]. According to Schulman A. et al. [43], only a 4% (*p* = 0.004) decrease in re-excision rates was observed after analyzing the ASBrS Mastery of Breast Surgery database in the post-SSO-ASTRO Consensus era. The results of our study strongly support the adoption of the current consensus on surgical resection margin for primary BCS, even in patients with aggressive molecular subtypes such as TNBC, but also encourage further studies for patients with upfront systemic therapy.

The limitations of our study are related to its retrospective design and single-center data collection. Moreover, the smaller number of patients could be seen as a flaw, but a long follow-up with detailed medical records and recent nature of our cohort should balance the quality of our analysis. Even though one-third of the studied patients had undergone neoadjuvant chemotherapy, we conducted a separate analysis for the NACT group in an attempt to minimize the errors of our study and find different possible outcomes for this cohort of patients. Furthermore, we conducted analyses for several cut-off values of surgical resection margin. To the best of our knowledge, this is the second study that specifically investigates the effect of surgical resection margin on LR in TNBC patients, but the first that analyzes LR according to several cut-off values of the surgical resection margin. Furthermore, as recommended in the SSO-ASTRO Consensus Guideline on Margins for Breast-Conserving Surgery (“the monitoring of outcomes at the institutional level is encouraged”) [18], this study also served as institutional analysis for the outcomes of a “no ink on tumor” approach.

Our study reiterates the scientific support for the “no ink on tumor” approach in BCS, even for patients diagnosed with TNBC. Moreover, it reflects the real-world data from a high-volume cancer center at the dawn of the neoadjuvant therapy era for breast cancer patients. Results point us toward new collaborative multicentric studies/analyses with large cohorts and trustworthy consensus statements regarding optimal surgical margin width for patients treated with upfront systemic therapy.

## 5. Conclusions

In conclusion, univariate and multivariate analyses failed to support the surgical concept of “wider is better” in our patients. Therefore, our results confirmed the safety of a “no ink on tumor” approach for BCS, even in patients diagnosed with TNBC.

This approach seems to be safe even for women treated with NACT, but caution and close monitoring are advised for this category. The new era of neoadjuvant therapy for TNBC urges further studies and analyses to reach an international consensus regarding surgical margin width for these patients.

## Figures and Tables

**Table 1:** Patients’ baseline characteristics according to surgical resection margin status.

Characteristics	Surgical Resection Margin Status		*p* Value
	≤2 mm*n* ^1^ (%)	>2 mm*n* (%)	
Age at surgery (years):			0.46
Mean ± SD ^2^	47.02 ± 7.93	50.15 ± 11.13	
		
<50	3 (42.86)	43 (50.59)	
≥3	4 (57.14)	42 (49.41)	
Menopause:			0.70
No	4 (57.14)	39 (45.88)	
Yes	3 (42.86)	46 (54.12)	
Clinical tumor size (cT):			0.66
T1 (≤2 cm)	1 (14.29)	24 (28.24)	
T2 + T3 (>2 cm)	6 (85.71)	61 (71.76)	
Clinical lymph-node status (cN):			0.44
Negative	5 (71.43)	44 (51.76)	
Positive	2 (28.57)	41 (48.24)	
Pathological tumor size (pT):			0.44
T1 (≤2 cm)	3 (42.86)	51 (60.00)	
T2 + T3 (>2 cm)	4 (57.14)	34 (40.00)	
Pathological lymph-node status (pN):			1.00
Negative	5 (71.43)	59 (69.41)	
Positive	2 (28.57)	26 (30.59)	
Lymphovascular invasion:			0.41
Absent	4 (57.14)	61 (71.76)	
Present	3 (42.86)	24 (28.24)	
Nottingham score:			0.44
I + II	2 (28.57)	40 (47.06)	
III	5 (71.43)	45 (52.94)	
Chemotherapy:			0.410.67
Neoadjuvant	1 (14.29)	32 (37.65)	
Adjuvant	6 (85.71)	58 (68.24)	

^1^*n*, number of patients; ^2^ SD, standard deviation.

**Table 2:** Number of local and distant recurrences according to surgical resection margin status.

Surgical Resection Margin Status	Recurrence		*p* Value for LR ^2^ DR ^3^
	Local *n* ^1^ (%)	Distant *n* (%)	
≤2 mm	0 (0.00)	0 (0.00)	1.00
>2 mm	5 (5.88)	8 (9.41)	1.00
≤3 mm	1 (9.09)	1 (9.09)	0.47
>3 mm	4 (4.93)	7 (8.64)	1.00
≤4 mm	1 (7.14)	2 (14.28)	0.57
>4 mm	4 (5.12)	6 (7.69)	0.35
≤5 mm	1 (3.57)	4 (14.28)	1.00
>5 mm	4 (6.25)	4 (6.25)	0.24
≤10 mm	2 (3.92)	5 (9.80)	0.65
>10 mm	3 (7.31)	3 (7.31)	0.72

^1^*n*, number of patients; ^2^ LR, local recurrence; ^3^ DR, distant recurrence.

**Table 3:** Summary of discussed studies investigating the risk of local recurrence after BCS ^1^ in TNBC ^2^ patients.

Author/Study	Study Type	Number of Included Breast Cancer Patients	Results
Lowery A.J. et al. [21]	Meta-analysis	12,592 7174 BCS 5418 mastectomy	Increased risk of LR ^3^ for TNBC patients
Houssami N. et al. [29]	Meta-analysis	14,571 14,571 BCS	Similar LR rates for different surgical margin widths (SMW) (1 versus 2 versus 5 mm; *p* > 0.10)
Pilewskie M. et al. [30]	Observational retrospective study	535 only TNBC 535 BCS	No difference in terms of LR (*p* = 0.11) and DR ^4^ (*p* = 0.53) for SMW ≤2 versus>2 mm
AdkinsF.C. et al. [38]	Observational retrospective study	1325 only TNBC 651 BCS 674 mastectomy	On multivariate analysis, close/positive margins were correlated with a higher LR risk

^1^ BCS, breast-conserving surgery; ^2^ TNBC, triple-negative breast cancer; ^3^ LR, local recurrence; ^4^ DR, distant recurrence.

## Data Availability

The data presented in this study are available on request from the corresponding author. The data are not publicly available due to ethical and institutional reasons.
